# Heterogeneity in public attitudes and preferences for the deployment of aquifer thermal energy storage

**DOI:** 10.1038/s41560-026-01977-z

**Published:** 2026-03-06

**Authors:** Ting Liu, Richard Hanna, Yiannis Kountouris

**Affiliations:** Centre for Environmental Policy, Imperial College London, London, UK

**Keywords:** Psychology and behaviour, Energy supply and demand

## Abstract

Aquifer thermal energy storage (ATES) can contribute to heating and cooling decarbonization by utilizing the thermal capacity of natural aquifers. Securing acceptance and support for deploying ATES at scale requires acknowledging public perceptions and designing systems compatible with public preferences. Here we characterize attitudinal stances and preferences for the deployment of ATES in public buildings in the UK. Using data from a social survey and a discrete choice experiment, we find substantial heterogeneity in public attitudes and support for ATES installations. Latent class analysis identifies four distinct stances, ranging from cautiously negative to enthusiastically supportive. Estimating mixed multinomial and hybrid choice models, we find strong preferences for quicker deployment of ATES infrastructure, with greater CO_2_ emissions-reduction capacity, that can be accessed by private households. Results point to the need for tailored communication strategies and preference-compatible design for achieving socially desirable sustainable energy transitions.

## Abstract (web summary)

Survey and discrete choice experiment data reveal substantial heterogeneity in the UK public’s stance towards aquifer thermal energy storage deployment and in preferences for deployment characteristics that vary with latent attitudes.

## Main

Decarbonizing heating and cooling is vital for the UK’s energy and net-zero strategies^1^. Heating accounts for approximately 40% of the country’s total energy use^2^ and contributes around 20% of its greenhouse gas emissions^3^. In 2021, space and water heating consumed approximately 663 TWh, with residential heating comprising 75% of this demand^4^. Whereas cooling currently amounts for a smaller proportion of energy use, this is projected to increase due to climate change and evolving building standards, further intensifying the need for the deployment of energy-efficient technologies^5^. Attempting to address the challenges associated with decarbonizing heating and cooling, the UK has set a target of installing 600,000 electric heat pumps annually by 2028 as part of its broader strategy to reach net-zero emissions by 2050^6^. Nevertheless, there is yet potential to expand the country’s heating and cooling technology portfolio taking advantage of hitherto unutilized resources to expedite the transition to sustainable low-carbon heat production^7^.

ATES utilizes underground aquifers to store thermal energy that can be later extracted and used for climate control^8,9^. By leveraging the thermal capacity of natural aquifers, ATES can meet urban heating and cooling needs sustainably, reducing dependence on fossil fuels and lowering carbon emissions. ATES is used across the world but more prominently in the Netherlands and Sweden where there are over 3,000 installations^10–12^. The UK has substantial potential for deploying ATES systems, owing to its favourable seasonal climate and the availability of suitable aquifers located near urban areas with high heating and cooling demands^13^. ATES can meet up to 61% of the UK’s heating demand and 79% of its cooling demand, reducing associated carbon emissions by 13–41% and 70–94%, respectively, when compared to traditional ground- or air-source heat pump systems^13^. As urban areas continue to expand and the demand for renewable energy solutions grows, the implementation of ATES can present a viable pathway towards a more sustainable energy future^14^.

Achieving widespread ATES adoption presents joint societal and design challenges. Unfamiliarity, mistrust and limited public acceptance have been significant barriers to the rollout of renewable technology infrastructure^15,16^, hindering the adoption of innovations that generate real or perceived local environmental impacts and landscape disamenities^17–21^. Acknowledging potential public concerns is necessary for ATES adoption and long-term viability. At the same time, evaluating the environmental benefits associated with the introduction of ATES is essential for approximating its full contribution to social well-being. In this context, understanding the public’s views towards ATES deployments, and their preferences over its characteristics, is important for informing their design and gaining community approval.

Much of the research on the attitudes and preferences towards renewable energy infrastructure has focused on its siting. Researchers have studied the drivers of “Not in my back yard” (NIMBY) and place-conservation behaviour^15,16,22^, and the local opposition that often arises due to lack of engagement, limited information availability^23^ and low awareness of the need for, and benefits to, decabonization^24,25^, which in turn lead to scepticism about the effectiveness of energy interventions. More recent contributions interpret opposition to energy infrastructure siting through an environmental justice lens^26–31^, suggesting that reaction may reflect concerns about distributive or procedural justice surrounding installation decisions. Furthermore, personal values prioritizing environmental conservation and sustainability, commitment to pro-environmental goals and normative social conventions appear as significant determinants of renewables acceptance and adoption^32,33^.

Research on the preferences for heating technology design characteristics primarily explores homeowners’ preferences over the distribution of costs in time^34,35^ and the environmental impacts of heating technology options^35–38^, using discrete choice experiments to study private-home installations where most of the associated costs and benefits directly accrue to property owners. More generally, an extensive literature looks at the public’s preferences and willingness to pay for renewable energy deployment characteristics^39–42^, focusing on wind power^43–46^, solar^47,48^, hydropower^49^ and biomass^50–53^. Evidence generally points to strong preferences for low-carbon energy^54^, with households and individuals deriving value from climate change mitigation^55^, local energy security and environmental improvements.

The public’s stance towards shallow geothermal solutions for heating and cooling and their preferences over design characteristics remain underexplored, especially in markets where such interventions are unfamiliar. Here we address this gap, studying public attitudes and preferences towards ATES deployment in the UK. We focus on installations aiming to heat and cool public-use buildings, drawing on data from a social survey and a discrete choice experiment applied on over 1,700 individuals in the northwest of England. Our results show substantial heterogeneity in attitudes, pointing to four distinct and diverging stances towards ATES deployment. We find that the public has strong, albeit heterogeneous preferences and willingness to pay for ATES deployment characteristics that vary with latent attitudes towards systemic and local benefits of ATES, the provision of ATES-related information, policy and financial support for ATES and concerns about safety and reliability. The paper contributes to the literature on public attitudes and preferences towards renewable energy adoption.

## Heterogeneous views on aquifer thermal energy storage

**Fig. 1:**
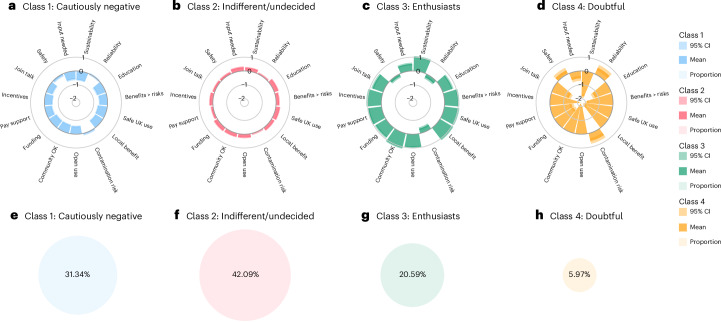
Predicted means and probabilities of the four-class ATES stance model. **a**–**d**, Predicted means and 95% confidence interval (CI) of class 1 (**a**), class 2 (**b**), class 3 (**c**) and class 4 (**d**) ATES attitudes. **e**–**h**, Predicted probabilities of class 1 (**e**), class 2 (**f**), class 3 (**g**) and class 4 (**h**) of ATES attitudes. Note that the predicted means of ATES attitudes refer to the model-estimated average levels of agreement with each attitude statement within each latent class. Predicted means greater than 0 indicate agreement, while means less than 0 indicate disagreement. Apparent darkening in some areas reflects the graphical overlap of the mean and the associated confidence interval. The attitude statements are as follows. Sustainability: ATES is a viable, sustainable solution for energy storage and supply. Reliability: I have concerns about ATES reliability and performance. Education: public education on ATES should increase. Benefits > risks: ATES benefits outweigh risks. Safe UK use: ATES should be deployed safely in the UK. Local benefit: ATES can benefit my local area’s sustainability. Contamination risk: ATES could risk groundwater contamination. Open use: I am open to using ATES for local heating/cooling. Community OK: I accept ATES systems in my community. Funding: governments should secure external funding for ATES. Pay support: I am willing to pay a small fee for local ATES projects. Incentives: I support incentives to encourage ATES adoption. Join talk: I am willing to join discussions on local ATES projects. Safety: I am concerned about ATES safety (for example, subsidence, pollution). Input needed: ATES needs environmental assessment and community input. Please be aware that this is a shortened version of the attitude statements. Full descriptions are detailed in the Methods section.

We explored the heterogeneity in the public’s views towards ATES, estimating a series of latent class models with increasing complexity (Methods). Our preferred specification identifies four latent classes. Figure 1 shows the predicted means for each latent attitude class represented by each Likert scale item and the associated predicted class membership probabilities.

Class 1 collects approximately 31.34% of the sample and comprises individuals with cautiously negative attitudes towards ATES. Whereas members do not outright reject ATES as a technological solution to an important problem, they are not fully convinced either. They have reservations about the technology’s long-term reliability and performance and are concerned over its possible environmental risks. They do not believe that ATES projects should undergo thorough environmental assessments and community consultations before being implemented at the local level, are doubtful of their overall benefits and tend to agree with statements linking ATES to risks of groundwater contamination, thermal pollution and subsidence, showing caution over perceived environmental threats of ATES technology.

Class 2 collects 42.09% of the sample, who appear to be indifferent or undecided regarding ATES deployment. Those respondents’ stance is relatively neutral, generally leaning towards agreement with positive statements but without strong conviction. For instance, their attitude towards ATES as a viable and sustainable heating and cooling solution indicates some level of support. They believe ATES has the potential to improve energy efficiency and sustainability in their local area but do not appear fully convinced. They tend to agree that ATES projects should undergo thorough environmental assessments and community consultations but as with the earlier statements, are not overly passionate about it.

Class 3 accounts for the enthusiasts who make up 20.59% of the sample. They are highly supportive and proactive in promoting ATES, seeing it as a critical solution for sustainable energy. They are strongly confident that the benefits outweigh the risks. They believe ATES could benefit their area in terms of energy efficiency and are open to deployments in their neighbourhood and local community. They support government incentives, are willing to contribute financially and are ready to actively participate in related community consultations to inform decision-making.

Class 4 collects the remaining 5.97% of the sample, who are doubtful of ATES implementation. This class is primarily concerned about the risks and negative impacts of ATES, showing little to no support for its deployment. Specifically, they disagree that ATES is a viable and sustainable solution, stressing the risks of groundwater contamination, subsidence and thermal pollution. Members are unconvinced that the benefits of ATES outweigh the potential risks. They oppose local ATES projects, demand thorough environmental assessments and community consultations, are unwilling to incur costs for ATES installations and sceptical about government incentives. When it comes to preferences of alternative renewable energies, class 4 opts to other solutions over ATES for long-term energy storage and stability.

**Fig. 2:**
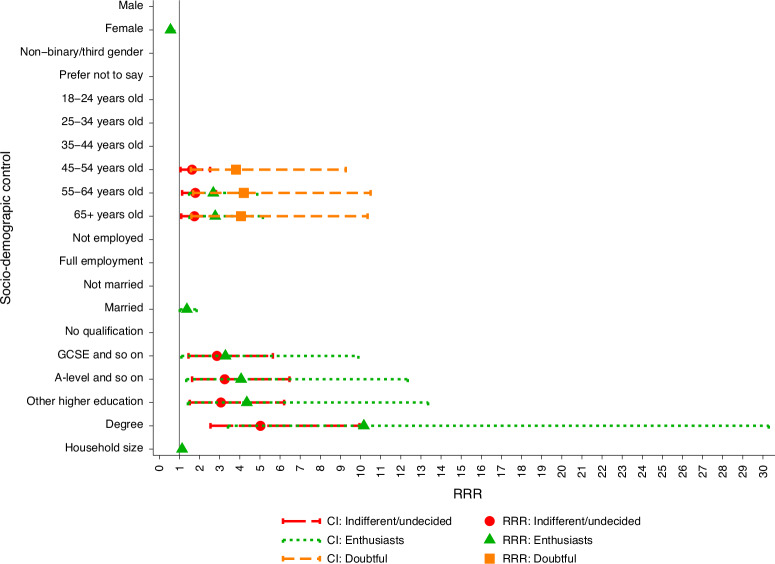
Relative risk ratio of socio-demographics on class membership. Class 1 (‘cautiously negative’) serves as the reference category. All coefficients are interpreted relative to their respective baseline categories. Note that only statistically significant relative risk ratios (RRRs) are presented to reduce visual clutter. An RRR of 1 is indicated by the solid vertical line. An RRR greater than 1 suggests a higher likelihood of belonging to a given class, whereas an RRR less than 1 indicates a lower probability. The horizontal dashed lines represent the 95% confidence intervals. GCSE and A-level denote standard academic qualifications in the UK, typically taken at ages 16 and 18, respectively.

Estimates from multinomial logistic regressions examining how class membership varies with a series of socio-demographic controls are reported in Fig. 2. The main results are also summarized in Supplementary Table 4. Age is consistently associated with attitude class membership. Across all age groups, the highest proportion of respondents in the ‘doubtful’ class (29.52%) belongs in the 65+ age band. By contrast, the highest proportion of ‘enthusiasts’ (20%) belong to the 16–24 age band, exceeding the proportion of any other class within that group. These results suggest that older respondents are significantly more likely to be ‘doubtful’ than ‘cautiously negative’, indicating greater uncertainty or reservations regarding ATES among older individuals. Higher education attainment increases the odds of belonging to the ‘enthusiasts’ class, suggesting that education can foster positive attitudes towards ATES and encourage support for local deployment. On the other hand, education attainment does not significantly differentiate between respondents in the ‘indifferent’ or ‘doubtful’ and ‘cautiously negative’ classes. Class membership appears to some extent gendered: men are significantly more likely to belong to the ‘indifferent’ class than the ‘cautiously negative’ reference class. However, gender does not significantly differentiate between ‘enthusiasts’ or ‘doubtful’ and the reference class. Married respondents show slightly higher odds of belonging to the ‘doubtful’ class than the ‘cautiously negative’ class, suggesting marriage might be associated with increased scepticism towards ATES. Large household size tends to increase the odds of respondents showing enthusiastic attitudes towards ATES, while employment status does not significantly predict differences in attitude class membership.

## Preferences over ATES deployment characteristics

We now turn to the results from mixed multinomial logit (MXL) models, using data from the discrete choice experiment, modelling the respondents’ utility from alternative ATES deployment scenarios as a function of ATES attributes. Column 1 of Table 1 shows estimates of the utility function in willingness to pay (WTP) space (Methods), using information from the full sample of 1,758 individuals. Attribute coefficients capture the mean of the marginal WTP distribution in GBP. The mean of the alternative specific constant for option 3 is negative and statistically significant, suggesting that respondents are in principle less likely to select the opt-out alternative relative to one of the two opt-in alternatives^56^. Respondents are on average willing to pay about £14 extra for ATES deployments allowing private household access, whereas their valuation of ATES increases by approximately £16 for every 1,000 tons of CO_2_ abated annually. Increasing the time to the project’s completion by a year lowers well-being by approximately £4. Finally, respondents are willing to pay about £0.8 to increase their distance from the ATES installations by 1 km. There is strong evidence of preference heterogeneity across respondents, indicated by the significant estimates of the standard deviations of parameter distributions.

**Table 1:** MXL in WTP space

Attribute	(1)		(2)	
	Mean	s.d.	Mean	s.d.
Option 3	−3.580***	4.494***	−2.486***	4.302***
	(0.164)	(0.198)	(0.333)	(0.198)
Time to completion (years)	−3.999***	4.219***	−4.285***	4.725***
	(0.275)	(0.210)	(0.292)	(0.219)
Private access	14.246***	20.509***	15.099***	19.188***
	(0.960)	(0.858)	(0.733)	(0.835)
CO_2_ emissions reduction (tons ×10^3^)	16.216***	15.321***	15.599***	16.756***
	(1.082)	(1.322)	(0.892)	(0.801)
Distance (km)	0.868***	1.733***	0.977***	0.956**
	(0.300)	(0.214)	(0.237)	(0.423)
Price (£)	3.377***	1.068***	3.384***	1.272***
	(0.046)	(0.053)	(0.053)	(0.055)
Female × option 3			0.229	
			(0.239)	
Employed × option 3			−0.741***	
			(0.243)	
Higher education × option 3			−1.330***	
			(0.242)	
Married × option 3			0.643***	
			(0.248)	
Household size × option 3			−0.186***	
			(0.071)	
Log-likelihood	−16,028.72		−15,997.47	
Akaike information criterion	32,081.45		32,028.95	
Individuals	1,758		1,758	
Observations	21,096		21,096	

Note that estimates from MXL models in WTP space using the full sample from the choice experiment application. The dependent variable is in all cases respondent choice between three profiles of ATES deployment. Option 3 is the opt-out alternative. Private access is binary, equal to 1 if the ATES deployment permits private household connections. Models are estimated using 5,000 Sobol draws. Column 1 shows estimates of the mean and s.d. of the WTP distributions for each attribute. Column 2 shows the same for a model including interactions with selected socio-demographic characteristics. Male, higher education, married and employed are all binary, equal to 1 for respondents identifying as male, with higher education, who are married and in employment, respectively. Standard errors in parentheses. ***P < 0.01, **P < 0.05, *P < 0.1.

Column 2 reports MXL estimates in WTP space when accounting for observed heterogeneity, interacting respondent socio-demographic characteristics with the opt-out alternative specific constant. Accounting for respondents’ sex, education, employment status, marital status and household size does not materially change results. Time-to-completion, private household access, CO_2_ emissions reduction and distance to the ATES installation remain significant determinants of choice, whereas WTP appears quantitatively similar to the model without interactions. As earlier there is strong evidence of preference heterogeneity suggested by the highly significant estimates of the standard deviations. Coefficients on the interaction terms suggest that individuals in full employment and with higher education are less likely to select the opt-out alternative. Disutility from the opt-out alternative appears to increase with household size but decreases for those that are in marriage. The Supplementary Information document discusses estimates from a multinomial logit (MNL) model in WTP space (Supplementary Table 5) that supplied the starting values for the MXL models and estimates from MNL and MXL models preference space (Supplementary Table 6) and the corresponding WTP values (Supplementary Table 7). Supplementary Tables 8 and 9 show estimates excluding individuals that consistently selected the opt-out alternative, with no change to the results.

To further explore whether latent attitudes influence choice, we use a hybrid choice model (HMXL) model in WTP space. As earlier we allow for heterogeneity assuming preferences are distributed randomly across respondents. For brevity, we present estimates from the choice component in Table 2, whereas Supplementary Tables 10 and 11 show the structural and measurement components, respectively. Estimates of means and standard deviations of the WTP distributions are qualitatively and quantitatively very similar to those derived from the MXL model. Respondents are willing to pay about £3.7 to reduce the time to deployment by one year and about £12 and £13 to have the opportunity to access the public ATES installation and reduce CO_2_ emissions by 1,000 tons, respectively. Nevertheless, the coefficient on the distance attribute is no longer statistically significant at conventional levels.

**Table 2:** Choice component of the hybrid MXL model

	(1)		(2)	(3)	(4)	(5)	(6)
			Interactions with latent variables				
	Mean	s.d.	LV1	LV2	LV3	LV4	LV5
Option 3	−2.287***	2.788***	−0.829***	−0.971**	−0.620***	0.597***	−0.638*
	(0.215)	(0.158)	(0.234)	(0.378)	(0.111)	(0.159)	(0.345)
Time to completion (years)	−3.698***	4.031***	0.137	−0.172	−0.487	1.084***	−1.314***
	(0.39)	(0.281)	(0.573)	(0.518)	(0.347)	(0.401)	(0.366)
Private access	12.213***	17.653***	6.505***	4.762**	−1.295	−0.234	1.139
	(1.474)	(0.985)	(1.614)	(2.116)	(1.373)	(1.676)	(1.347)
CO_2_ emissions reduction (tons ×10^3^)	12.524***	12.861***	3.605	2.376	−4.300**	8.291***	−1.141
	(1.979)	(1.916)	(2.854)	(2.564)	(1.765)	(1.421)	(1.648)
Distance (m)	0.485	0.631	−1.328	0.164	−0.876	1.632***	0.724
	(0.481)	(0.566)	(0.985)	(0.651)	(0.546)	(0.463)	(0.845)
Price (£)	3.250***	1.000***	0.425***	0.106	−0.116**	−0.473***	0.200***
	(0.08)	(0.06)	(0.083)	(0.108)	(0.053)	(0.053)	(0.061)
Log-likelihood	−47,822.45						
Akaike information criterion	95,928.9						
Individuals	1,758						
Observations	21,096						

Note that the table presents estimates of the choice component from a hybrid choice MXL in WTP space. The dependent variable is in all cases respondent choice between three profiles of ATES deployment. Option 3 is the opt-out alternative. Private access is binary, equal to 1 if the ATES deployment permits private household connections. Column 1 shows estimates of the mean and s.d. of the WTP distributions for each attribute. Columns 2–6 show estimates of the coefficients on the interaction terms between the attributes and each of the latent variables. The latent variables capture attitudes towards: systemic benefits of ATES (column 2), local and individual benefits of ATES (column 3), safety and environmental concerns (column 4), community engagement and education (column 5), policy and financial support (column 6). Standard errors in parentheses. The model is estimated using 3,000 Sobol draws. ***P < 0.01, **P < 0.05, *P < 0.1.

We now turn to the latent variable interaction terms, capturing the relationship between ATES attributes and the unobserved attitudes. All coefficients on the option 3 × LV terms are statistically significant. Individuals with greater expectation of system-wide (LV1) and local-private (LV2) benefits from ATES, greater concern for sustainability and safety (LV3) and stronger support for policy and financial assistance to the technology (LV5), experience greater disutility from choosing the opt-out alternative. On the other hand, greater support for education initiatives (LV4) is positively related to the utility from the status quo alternative.

Greater expectations of system-wide benefits (LV1), and local or individual benefits of ATES (LV2) appear to increase WTP for private access. On the other hand, greater concern over the risks and sustainability of ATES (LV3) is related to lower WTP for CO_2_ emissions reduction. Greater support for ATES-related information initiatives (LV4) is related to greater utility from CO_2_ emissions reduction. At the same time, it is linked to lower disutility from increasing the time to deployment and greater utility from increasing the distance to the installation. Finally, more positive stance towards policy and financial support for ATES increases the disutility from delaying deployment. Supplementary Tables 12–14 show qualitatively and quantitatively similar results from an HMXL model when excluding respondents who consistently choose the opt-out alternative.

## Discussion and conclusion

The transition to efficient low-carbon heating and cooling is essential for addressing decarbonization challenges and meeting ambitious net-zero targets. Subsurface thermal storage capacity provides a natural resource that can be harvested to this end through ATES systems. Designing effective ATES deployments in a scalable and responsible manner that minimizes public opposition and maximizes social benefits requires understanding the public’s attitudes, concerns and preferences.

Drawing on information from a social survey and a discrete choice experiment conducted in England, we characterized substantial heterogeneity in respondents’ stance towards ATES and preferences for its characteristics. Close to 40% of our sample show some scepticism and tend to oppose local ATES deployments. These ‘cautiously negative’ and ‘doubtful’ respondents are unconvinced about the long-term reliability and are concerned over environmental risks of ATES. They appear worried about the possibility of groundwater contamination and the risks of subsidence. About 42% of the sample are indifferent or undecided, maintaining a neutral stance, showing slight support for deployment but lacking conviction. Approximately 21% are Enthusiasts demonstrating strong support for ATES as a sustainable heating and cooling solution, endorsing government incentives and community involvement. Estimates from mixed multinomial logit models in WTP space show strong evidence that the public values quick development, the associated carbon emissions mitigation and the opportunity to use ATES for private-home heating. There is some evidence that the public would prefer to increase their distance between ATES systems and their properties, but this result is not stable across models.

Our analysis points to significant correlations between latent attitudes towards ATES and preferences for its attributes. Results suggest that the choice between the opt-in and opt-out alternatives is influenced by considerations of collective interest. Latent attitudes over systemic benefits, policy support, safety and information influence the respondents’ willingness to endorse ATES in principle. This implies that choice may be also contingent on normative evaluations of the distribution of benefits, risk and information^56,57^.

Individuals with greater latent support for community engagement and information tend to select the opt-out alternative relative to the opt-in options. We interpret this as suggestive of risk aversion^58^ and reluctance towards the technology. These individuals acknowledge that awareness of the technology is limited and require more information to be convinced of its effectiveness. In contrast, expectations of systemic and local benefits introduce aversion to inaction, rendering non-adoption undesirable, encouraging respondents to select one of the opt-in alternatives.

Findings can be further interpreted through the joint lenses of the expectation-confirmation^59^ and value-belief-norm^60^ theories. Our results suggest that more positive attitudes towards policy and financial support for ATES are associated with greater disutility from longer time to deployment. It is reasonable to assume that individuals holding supportive views towards ATES will tend to expect its timely deployment. When deployment is delayed, the distance between expectation and outcome increases, implementation appears ineffective, leading to disappointment and lower utility.

At the same time, we find that greater latent support for community engagement and information is related to lower disutility from pushing deployments further into the future. Individuals supporting engagement and information activities implicitly acknowledge their limited understanding of the technology and believe that its successful implementation requires public support that can be achieved through information dissemination and engagement. They do not expect rapid deployment and consequently are not disappointed when development time increases. Instead, they are motivated to support information provision and are likely to declare their preference for alternatives that allow for it through a longer deployment horizon.

Results suggest that greater expectation of local and systemic benefits is positively related to WTP for securing private access to the system. This is expected, as individuals who anticipate personal and social gains from ATES, will tend to be more confident that their contribution will yield valuable outcomes, increasing their motivation to pay for access.

Concerns over the safety of ATES are linked to lower willingness to pay for CO_2_ emissions reduction. This reflects a trade-off between trust in the technology and pro-environmental behaviour^54^. It suggests that perceived risks associated with subsurface technologies can impose an environmental cost by reducing public support for climate change mitigation efforts. On the other hand, we find that greater support for community engagement and information is associated with stronger preferences for CO_2_ emissions reduction. This plausibly indicates that information-seeking individuals are motivated by the pro-environmental function of ATES. Finally, we find that stronger support for community engagement and information is related to higher WTP for increasing the distance to the ATES installation. As argued earlier, this probably reflects unfamiliarity, uncertainty and risk aversion on the behalf of respondents.

Findings agree with prior research suggesting that the public values the environmentally improving function of green and renewable energy systems^35,37,42^. Respondents acknowledge the impact of heating and cooling on the national carbon footprint and are amenable to shouldering part of the cost for reducing it, even though their preferences vary substantially and depend on latent attitudes. This is encouraging for the potential of integrating ATES systems into existing urban infrastructure and hints that the public acknowledges the need for innovative technological solutions for climate change mitigation. Nevertheless, our results point to potentially significant barriers that ATES developers and policymakers may need to address. Nearly 40% of respondents are to some extent hesitant and sceptical over the technology’s potential, whereas another 40% appear unconvinced. We speculate that these respondents are influenced by ongoing debates regarding the environmental impacts of shale gas extraction^61,62^.

The results point to a path that policymakers and professionals could follow for the effective development of low-carbon heating and cooling interventions in general and ATES in particular. Familiarizing the public with the features of the new technology is essential to mitigate against misconceptions about risk and performance. This is particularly important given that ATES as subsurface technology can be misunderstood to belong to the family of fracking interventions^63^ that attract considerable opposition.

The substantial willingness to pay for private access to public ATES systems points to opportunities for the development of urban district heating networks^64^. Furthermore, the distributed nature of such systems can trigger greater consumer participation and involvement in the energy market^25,63^.

The public appears to favour reducing CO_2_ emissions through ATES. This suggests that widespread acceptance can be gained when ATES is presented as part of a climate change mitigation strategy. Furthermore, respondents’ preference for rapid deployment may also reflect the urgency perceived by the public in addressing environmental issues. Aligning ATES deployment strategies with community expectations, particularly emphasizing CO_2_ reduction benefits, may enhance support, especially from groups that show enthusiasm or moderate support. This agrees with previous research suggesting that the use of ATES can be balanced and optimized more cost effectively by integrating it in housing, commercial and industrial developments^65^.

Despite rapid innovation and growth in the ATES industry internationally, public awareness and understanding of ATES remain limited in the UK. To increase public awareness, social acceptance and eventually the effectiveness of ATES development, communication strategies and deployment features should reflect the strong heterogeneity of public preferences. Insights obtained here can contribute to this direction, improving the adoption of ATES technology and supporting the broader UK energy decarbonization strategy.

## Methods

### Data

Data were collected through a survey instrument designed to elicit information about individual attitudes and preferences for the deployment of ATES in the northwest of England^66^. The survey was administered online on a sample of 1,758 respondents that were representative of the UK population. Respondents were recruited from a panel provided by Norstat, a major third-party survey recruitment company. Respondents were selected via quota sampling with regionally representative quotas for location, gender, age and level of education. Our analysis was restricted to adults that lived in a council-tax-paying household. Only respondents that fully completed the survey were included in the sample. Ethics approval was sought and granted by the Imperial College Ethics Board (SETREC number: 6799602). Sample descriptive characteristics are presented in Supplementary Table 1.

The survey comprised three parts. In the first part, respondents were offered background information regarding the UK’s commitments for decarbonization and the role of heating and cooling in the country’s carbon footprint. They were then introduced to a brief technical summary of ATES, describing the basic scientific and engineering principles underlying the operation, development and deployment of ATES. Respondents were then presented with a series of questions designed to elicit their familiarity with, understanding of and interest in ATES and its potential applications. Specifically, we focused on respondents’ attitudes towards (1) the system-level and (2) the local and individual-level impact of ATES, (3) respondents’ safety and environmental concerns, (4) their attitudes towards ATES-related community engagement and education, and (5) their attitudes on policy and financial support of ATES. These items, presented in Supplementary Table 15, were the objects of the latent class analysis described later. Participants indicated their agreement with each statement using a 5-point Likert scale (in which 1 denotes strongly disagree, 5 denotes strongly agree).

The second part of the survey contained the discrete choice experiment application^67^. We started by presenting respondents with a hypothetical local ATES deployment policy. We asked respondents to consider that their local (municipal) authority is planning to deploy a series of ATES installations as part of their efforts to sustainably heat and cool a portfolio of public-use buildings including schools, hospitals, and offices. Following consultations with stakeholders and experts involved in ATES development^68^, we described the proposed ATES interventions in terms of the following attributes. (1) Time to deployment. This attribute intends to capture preferences over the speed of ATES development. Respondents were instructed that depending on the technical specifications of the deployment, subsurface conditions and permits issuance, the proposed ATES systems would be online in 2, 3, 4 or 6 years from the time of the survey. (2) Private household access. This attribute intends to quantify preferences over the potential private benefits that could accrue to households. We instructed respondents that the proposed ATES system may or may not be able to accommodate private household connections through the creation of district heating and cooling systems. (3) CO_2_ emissions reduction. The attribute aims to capture preferences over the environmental benefits of ATES deployment, especially in the context of climate change mitigation. Respondents were informed that depending on the technical specifications of installations, CO_2_ emissions would be reduced by 150, 300, 500 or 1,000 tons per year for the next 20 years. To provide a point of reference, we informed them that on average a UK household emits about 10 tons of CO_2_ per year. (4) Distance to nearest ATES installation. This attribute intends to capture the disutility from the disruption associated with nearby ATES development^69^. Proximity could be 100 m, 500 m, 1 km or 3 km. (5) Cost. ATES deployment would be financed through a one-off payment levied to all council-tax-paying households. The additional payment could be £5, £10, £15, £20 or £35. The payment vehicle was selected on the basis of its familiarity and credibility. At the time of writing, council tax rates in the area ranged from £1,300 to £4,200. Although the proposed ATES payment is small compared to the total council tax paid, it would be in addition to the usual 3–5% annual increase. With an installation cost of up to £3 million and around 200,000 council-tax-paying households, the proposed contributions are sufficient to cover the expense. The choice experiment alternatives were constructed using a *D*-efficient experimental design (*d*-error = 0.0135) optimized for a multinomial logit model. Following the attributes’ description, respondents were presented with 12 choice cards, each containing two opt-in alternatives and an opt-out alternative. Supplementary Fig. 5 presents an example choice card. Respondents were instructed to select their most preferred among the three alternatives, while they were reminded of their budget constraint and payments they make for similar goods and services.

The final part of the survey collected standard socio-demographic information, including respondents’ age, gender, educational attainment, employment status, marital status, household size and income in bands.

### Latent class analysis

We used latent class analysis (LCA)^70^ to generate latent groups of attitudes towards ATES and study the role of socio-demographic characteristics in determining class membership. Latent classes are unobserved groups of individuals who share similar characteristics, defined by categories of a nominal latent variable. LCA estimates the likelihood that an individual belongs to a specific class based on their responses to a set of questions regarding understanding of ATES and assigns them accordingly. The membership probability reflects the likelihood that an individual belongs to a particular class based on their responses to the indicators^71^. Noted that as an inherently probabilistic approach, LCA estimates the posterior probability of class membership for each individual rather than providing deterministic assignment. For analytical interpretation, individuals were allocated to the class associated with the highest posterior probability; however, it is acknowledged that classification uncertainty persists owing to potential measurement error in both the observed indicators and the latent assignments.

Producing latent groups allows for a concise representation of public attitudes towards ATES and the underlying concerns over environmental and financial issues, safety and local community benefits. Technically, classification of attitudes treats latent constructs as categorical rather than continuous, leading to parsimony and dimensionality reduction when capturing variance within and describing a population^72^. LCA is an effective way to probabilistically identify homogenous groups within data, suitable for small- or medium-sized samples owing to its ability to analyse both categorical and interval-scale variables and its capacity to include cases with missing data^73,74^. Unlike other classification techniques such as cluster analysis or *k*-means clustering, LCA is model-based and permits a mathematical evaluation of how well a proposed LCA model represents the data^75^.

We started with a two-class model including 15 standardized attitudes variables and hypothesized 15 classes exist. The latent profile model treats original and standardized variables the same based on sensitivity analysis. We chose to present the results with standardization for theoretically better interpretation. With standardized variables, means above 0 means agree and below 0 means disagree. The selection of the optimal class solution was based on model fit using the Akaike information criterion (AIC) and Schwarz’s Bayesian information criterion (BIC). The BIC and AIC were minimized for the 12 and 15 latent class models respectively (Supplementary Table 2 and Supplementary Fig. 1). However, it is unrealistic to interpret 12 or 15 classifications, and the AIC values for a class greater than 15 could be smaller than those for 15. Earlier work introduces the concept of using an elbow plot of fit statistics to examine model fit^75^. Therefore, we plotted the values of AIC and BIC to find the ‘elbow’ of the point of diminishing returns in model fit (Supplementary Fig. 1). Small decreases in the AIC and BIC for each additional latent class were observed for the four and seven-class models, suggesting either of these models are viable options. We decided to retain the four-class specification as the estimated parameters are mostly stable across the various specifications: the structure of the model is reasonably robust and provides interpretable insights into varying attitudes patterns of ATES. Our attempts to estimate a model with five or six latent classes resulted in the fifth and sixth class collapsing into the fourth one. We further tested several latent class specifications with class-specific parameters for the sensitivity to the predicted means and class membership estimated as a simple probability. Finally, we tested several latent class models (for example, three-class, four-class and five-class) with class-specific parameters for sensitivity but including appropriate class membership probabilities and class membership functions. This enabled us to investigate the demographic characteristics of the respondents belonging to each class.

### Discrete choice experiment

To analyse the DCE data we employed MXL and HMXL models mixed multinomial logit models (MXL) and mixed hybrid choice models (HMXL) to account for unobserved individual preference heterogeneity, and in the case of the HMXL to gain further intuition on the relationship between attitudes, and preferences for ATES. In all cases, we estimate the utility function in WTP space, guaranteeing that WTP distributions have finite moments^76^. On the basis of the random utility theory^77^, we model the utility respondent *i* receives from option *j*, in choice occasion *n* as:

1 $${U}_{{ijn}}={V}_{{ijn}}+{\epsilon }_{{ijn}}$$

where ${V}_{{ijn}}$ and ${\epsilon }_{{ijn}}$ are the deterministic and random components of utility, respectively. We assume the deterministic component of utility is a linear function of ATES attributes:

2 $${U}_{{ijn}}=\beta {\prime} {X}_{{ijn}}+{\epsilon }_{{ijn}}$$

where ${X}_{{ijn}}$ is a matrix of attributes (time-to-completion, private household access, CO_2_ emissions reduction, distance and price). Assuming that the random component follows a Gumbel, Type 1 extreme value distribution obtains the multinomial logit model suggesting that the conditional choice probability for alternative *j* is given by:

3 $$P\left({y}_{{in}}=j,|,X\right)=\frac{\exp ({\beta }^{{\prime} }{X}_{{ijn}})}{{\sum }_{t=1}^{J}\exp (\beta {\prime} {X}_{{itn}})}$$

Given that respondents make a sequence of *N* choices, the conditional probability of a sequence of observed choices becomes:

4 $$P\left({y}_{i}=j,|,X\right)=\mathop{\prod }\limits_{n=1}^{N}\frac{\exp (\beta {\prime} {X}_{{ijn}})}{\mathop{\sum }\nolimits_{t=1}^{J}\exp (\beta {\prime} {X}_{{itn}})}$$

The mixed logit model extends the MNL model incorporating preference heterogeneity by assuming that preferences (parameters *β*) vary across individuals following some distribution $\beta \sim f(\bar{\beta ,}\Theta )$. Equation (4) then becomes:

5 $$P\left({y}_{i}=j,|,X\right)=\int \mathop{\prod }\limits_{n=1}^{N}\frac{\exp \left(\beta {\prime} {X}_{{ijn}}\right)}{\mathop{\sum }\nolimits_{t=1}^{J}\exp \left(\beta {\prime} {X}_{{itn}}\right)}f\left(\beta ,|,\Theta \right)d\beta$$

As is common in the literature, we assume that coefficients on non-monetary attributes follow a normal distribution while the coefficient on price follows a negative lognormal distribution.

Equation (2) can be parametrized in WTP space as:

6 $${U}_{{ijn}}={\alpha }_{{ij}}+{\psi }_{i}\left({\delta }_{i}^{{\prime} }{X}_{{ijn}}+{pric}{e}_{{ijn}}\right)+{\epsilon }_{{ijn}}$$

where coefficients ${\delta {\prime} }_{i}$ are interpreted as the willingness to pay for the corresponding attributes. ${\alpha }_{{ij}}$, ${\delta {\prime} }_{i}$ and ${\psi }_{i}$ are individual specific coefficients to be estimated.

Hybrid choice models (HCMs) incorporate latent variables capturing unobserved psychological and attitudinal individual characteristics in the discrete choice modelling framework. While unobserved, these latent variables are assumed to be correlated with a set of observable indicators that are typically recorded in an ordered scale through a series of Likert questions. HCMs typically comprise three components. The structural component of the HCM that connects latent variables with a series of socio-demographic characteristics is described by:

7 $${\mathrm{LV}}_{i}=\gamma {\prime} {Z}_{i}+{\eta }_{i}$$

where ${\mathrm{LV}}_{i}$ is the latent variable for individual *i*, ${Z}_{i}$ is a matrix of socio-economic characteristics and ${\eta }_{i}$ is a random term that follows a standard normal distribution to allow identification^78–80^.

The measurement component links the latent variable to observable attitudinal indicators, often captured by ordered variables derived from Likert scale questions:

8 $${I}_{i}=\zeta {\prime} {\mathrm{LV}}_{i}+{u}_{i}$$

where $\zeta {\prime}$ is a vector of coefficients capturing the correlation between the latent variable and the attitudinal indicator ${I}_{i}$ and ${u}_{i}$ is an error term. In our application each latent variable is assumed to be related to three indicators each, presented in Supplementary Table 3. As indicators are reported on a 5-point Likert scale, the measurement component for each latent variable is represented through a series of ordered logits.

The choice component of the HMXL augments equation (6) incorporating the latent variables through interaction terms with all attribute coefficients and the alternative specific constant so that:

9 $${\beta }_{i}=\Lambda {\prime} {\mathrm{LV}}_{i}+{{\beta }_{i}}^{* }$$

where ${\Lambda }^{{\prime} }$ and ${{\beta }_{i}}^{* }$ coefficients to be estimated. All choice models were estimated in R using the Apollo package^81^.

### Robustness using *k*-means clustering

We conducted robustness tests of ATES attitudes classifications using *k*-means clustering. *k*-means cluster analysis is used to group people’s attitudes into homogeneous clusters. In contrast to latent class analysis, *k*-means cluster is a centroid-based algorithm that assigns observations to clusters by minimizing the distance between each data point and its corresponding cluster centroid, thereby reducing overall within-cluster dispersion. To identify the most appropriate number of clusters, scree plots were examined for solutions ranging from one to 20 clusters, using both the within sum of squares (WSS) and its logarithmic transformation, log(WSS)^82^. The optimal solution was indicated by an elbow point, beyond which further increases in the number of clusters yield only marginal reductions in within-cluster variability.

To further evaluate model fit, the *η*^2^ coefficient was calculated to quantify the proportion of total variance explained by a given cluster solution, expressed as the reduction in WSS relative to the total sum of squares (TSS):

10 $${\eta }^{2}=\frac{\mathrm{WSS}(k)}{\mathrm{TSS}}$$

In addition, the proportional reduction of error (PRE) coefficient was employed as a complementary criterion to assess the improvement of a *k*-cluster solution over the preceding solution with *k* − 1 clusters^83^.

11 $${\mathrm{PRE}}_{k}=\frac{\mathrm{WSS}\left(k-1\right)-\mathrm{WSS}(k)}{\mathrm{WSS}(k-1)}\forall k\ge 2$$

To reduce the risk of convergence to suboptimal local minima, the clustering procedure was repeated 50 times using randomly initial cluster centroids, following established practice in a previous study that applied *k*-means clustering to attitudes towards climate change risks^84^. Following model estimation, clusters were interpreted and labelled based on clear and systematic differences in attitudes towards ATES as reflected in the cluster centroids. In total, 1,758 people were grouped into different discrete clusters based on their agreement on 15 statements about ATES benefits, risks and impact. The *k*-means results showed that a four-cluster solution provided the optimal classification. At *k* = 4, a kink or cut-off point was observed in both the WSS and log(WSS) figures, suggesting that additional clusters produced only negligible gains in explanatory power (Supplementary Figs. 2 and 3). Supplementary Fig. 4 shows similarity in four-class pattern to the latent class analysis. Therefore, *k*-means clustering robustly verified the classification derived from the latent class analysis, demonstrating consistency and confirming that respondents can reliably be grouped into four distinct segments based on their responses of ATES statements.

### Reporting summary

Further information on research design is available in the Nature Portfolio Reporting Summary linked to this article.

## Supplementary information

Supplementary Information Supplementary Figs. 1–5 and Supplementary Tables 1–15.

Reporting Summary

## Data Availability

The data are available via Figshare at 10.6084/m9.figshare.30762362 (ref. ^66^).

## Code availability

Code used to produce the results is available via Figshare at 10.6084/m9.figshare.30762362 (ref. ^66^).
