# Supplementary material for: Heterogeneity in public attitudes and preferences for the deployment of aquifer thermal energy storage
Source: Nat Energy. 2026 Mar 6;11(3):479–89. doi: 10.1038/s41560-026-01977-z (PMC13021514; doi:10.1038/s41560-026-01977-z)
Supplement: Supplementary file 1 — Supplementary Figs. 1–5 and Supplementary Tables 1–15. [file 41560_2026_1977_MOESM1_ESM.pdf]

nature energy

Supplementary information

https://doi.org/10.1038/s41560-026-01977-z

Heterogeneity in public attitudes and preferences for the deployment of aquifer thermal energy storage

In the format provided by the authors and unedited

Supplementary Information

This document presents supplementary information for the paper "Heterogeneity in public attitudes and preferences for the deployment of aquifer thermal energy storage".

1. Sample descriptive characteristics

Table 1 presents the main demographic characteristics of our sample. Among the 1758 respondents surveyed, 53.7% were female and 45.68% were male. Nearly 40% of the respondents were between the ages of 25 and 44. 52.9% had completed at least higher education, nearly half of the sample were employed full-time (48.86%), and a similar proportion (48.75%) were married. About 89% of respondents lived in households with fewer than five people.

Table 1. Descriptive statistics of socio-demographic characteristics of the respondents.

**Socio-demographic variable**	**Category**	**N**	**Percentage**
Gender	Male	803	45.68
	Female	944	53.7
	Non-binary / third gender	7	0.4
	Prefer not to say	4	0.23
Age	18–24	209	11.89
	25–34	368	20.93
	35–44	314	17.86
	45–54	267	15.19
	55–64	288	16.38
	65+	312	17.75
Educational attainment	Degree	690	39.25
	Other higher education	240	13.65
	A-level etc	354	20.14
	GCSE etc	418	23.78
	No qualification	56	3.19
Employment status	In full time employment	859	48.86
	In part-time employment	299	17.01
	Retired	316	17.97
	Looking for employment	79	4.49
	Student	53	3.01
	Houseperson	145	8.25
	On maternity/paternity	7	0.4
Marital status	Not in Married	901	51.25
	Married	857	48.75
Household size	1	323	18.37

	2	577	32.82
	3	356	20.25
	4	311	17.69
	5	105	5.97
	6	45	2.56
	7	11	0.63
	8	4	0.23
	9	6	0.34
	10+	20	1.15
Total		1758	100

Note: Household size refers to the total number of individuals living in the household.

2. Additional results from the LCA

Table 2 shows the AIC and BIC criteria for the latent class model estimated with different number of classes. The model with 12 latent classes has the smallest BIC and 15 latent classes has the smallest AIC would be considered the best based on these information criteria. However, it is unrealistic to interpret 12 or 15 classifications, and the AIC values for a class greater than 15 could be smaller than those for 15. Hence, we used an elbow plot of fit statistics to find the “elbow” of point of the “diminishing returns” in model fit.

Table 2. Model fit of different latent class models with full sample (N=1758).

Latent Class Model	N	Log likelihood (null)	Log likelihood (model)	*df*	AIC	BIC
2	1,758	.	-33779.9	46	67651.71	67903.41
3	1,758	.	-32913.2	62	6595.29	66289.55
4	1,758	.	-3166.9	78	63477.77	63904.58
5	1,758	.	-31321.1	94	6283.11	63344.47
6	1,758	.	-31085.5	110	62391.07	62992.98
7	1,758	.	-30648.8	126	61549.50	62238.96
8	1,758	.	-30569.1	142	61422.12	62199.13
9	1,758	.	-30377.5	158	6107.89	61935.46
10	1,758	.	-30258.1	174	60864.14	61816.26
11	1,758	.	-30196.7	190	60773.45	61813.11
12	1,758	.	-30082.3	206	60576.57	***61703.79***
13	1,758	.	-30029.1	222	60502.26	61717.03
14	1,758	.	-30089.2	238	60654.37	61956.69
15	1,758	.	-2995.8	254	***60409.60***	61799.47

Note. The lowest value of BIC and AIC were bold and italic.

Figure 1 visualises the values of the AIC and BIC for each latent class model. Small decreases in the AIC and BIC for each additional latent class were observed for the four and seven-class models, suggesting these either of these models are viable options. Therefore, we estimated both four-class and seven-class model to compare class pattern and class probabilities between the two models. We take the view that the four-class model offers clearer insight than the seven-class model and hence is most suitable for present purposes.

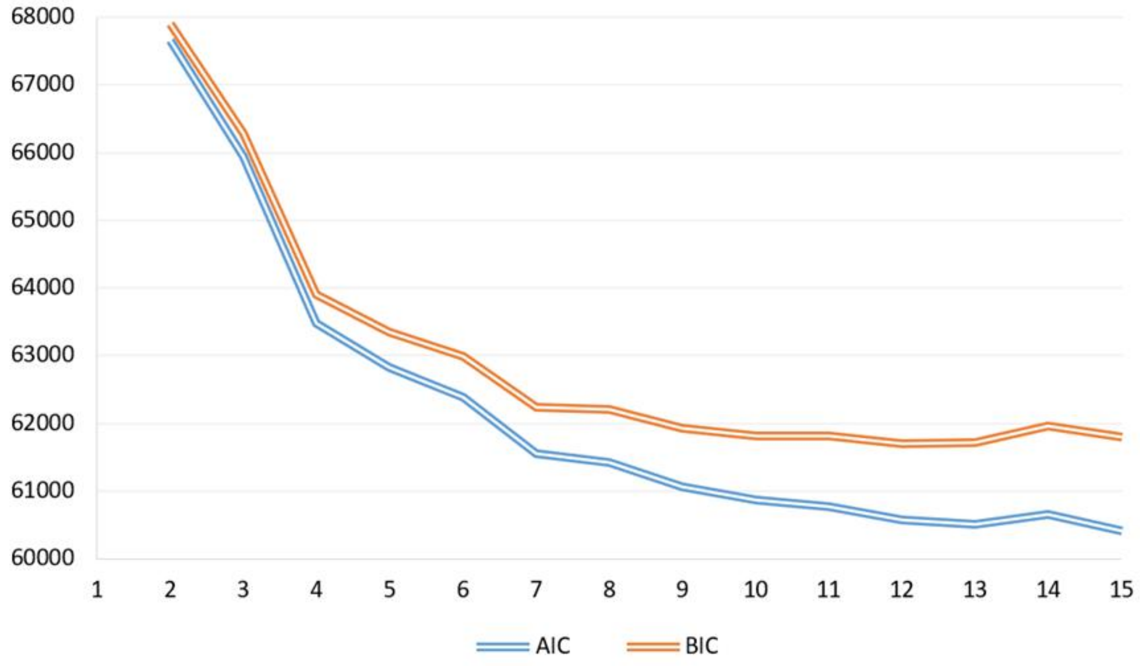

Figure 1. Values of Information criteria for latent class models with two to fifteen-class models (N=1758).

Table 3 shows the descriptive statistics for the attitudinal variables used in the latent class analysis. Responses of ATES attitudes were measured on a 5-point Likert scale ranging from 1 ("strongly disagree") to 5 ("strongly agree"). Overall, the mean scores for most attitude statements range from approximately 3.2 to 4.1, suggesting that participants generally expressed moderate to high agreement with the ATES-related attitudes.

Table 3. Descriptive statistics of 15 ATES attitudes variables.

Variable	Obs	Mean	Std. dev.	Min	Max
Sustainability	1,758	3.60	.98	1	5
Reliability	1,758	3.33	1.00	1	5
Education	1,758	3.98	1.03	1	5
Benefits > Risks	1,758	3.45	1.03	1	5
Safe UK use	1,758	3.73	.99	1	5

Local benefit	1,758	3.65	.98	1	5
Contamination risk	1,758	3.27	.95	1	5
Open use	1,758	3.60	1.00	1	5
Community OK	1,758	3.67	1.01	1	5
Funding	1,758	3.74	.98	1	5
Pay support	1,758	3.23	1.20	1	5
Incentives	1,758	3.59	1.02	1	5
Join talk	1,758	3.49	1.08	1	5
Safety	1,758	3.56	1.00	1	5
Input needed	1,758	4.08	.99	1	5

Note. The attitude statements are as follows: *Sustainability*: ATES is a viable, sustainable solution for energy storage and supply. *Reliability*: I have concerns about ATES reliability and performance. *Education*: Public education on ATES should increase. *Benefits > Risks*: ATES benefits outweigh risks. *Safe UK use*: ATES should be deployed safely in the UK. *Local benefit*: ATES can benefit my local area's sustainability. *Contamination risk*: ATES could risk groundwater contamination. *Open use*: I am open to using ATES for local heating/cooling. *Community OK*: I accept ATES systems in my community. *Funding*: Governments should secure external funding for ATES. *Pay support*: I am willing to pay a small fee for local ATES projects. *Incentives*: I support incentives to encourage ATES adoption. *Join talk*: I am willing to join discussions on local ATES projects. *Safety*: I am concerned about ATES safety (e.g., subsidence, pollution). *Input needed*: ATES needs environmental assessment and community input.

Table 4 shows the relative risk ratios of class membership by socio-demographics and percentage of belonging to each ATES attitudes class.

Table 4. Multinomial logistic regression model for four-class ATES attitudes.

Co-variates	Class 1: Cautiously negative		Class 2: Indifferent/ Undecided		Class 3: Enthusiasts		Class 4: Doubtful	
	RRR	%	RRR	%	RRR	%	RRR	%
Gender								
Male	-	41.02	1	42.97	1	56.63	1	51.43
Female	-	58.08	.93	56.62	.55***	42.54	.70	48.57
Non-binary	-	.36	1.30	.41	2.07	.55	.00	-
Prefer not to say	-	.54	.00	-	.43	.28	.00	-
Total	-	100	-	100	-	100	-	100
Age								
18-24 years old	-	15.43	1	11.76	1	8.01	1	7.62
25-34 years old	-	21.05	1.33	22.16	1.40	22.10	.78	7.62
35-44 years old	-	19.78	1.01	15.81	1.66	22.65	.60	5.71
45-54 years old	-	14.16	1.63*	16.08	1.67	12.43	3.81**	23.81
55-64 years old	-	13.79	1.79*	16.76	2.68**	16.85	4.20**	25.71
65+ years old	-	15.79	1.74*	17.43	2.78**	17.96	4.06**	29.52
Total	-	100	-	100	-	100	-	100
Employment								
Not in employed	-	51	1	53	1	43	1	67
Full employment	-	49	.86	47	1.07	57	.66	33
Total	-	100	-	100	-	100	-	100

Marriage status								
Not in married	-	57.35	1	52.16	1	39.78	1	52.38
Married	-	42.65	1.04	47.84	1.37*	6.22	.92	47.62
Total	-	100	-	100	-	100	-	100
Education								
No qualification	-	5.81	1	1.89	1	1.10	1	5.71
GCSE etc	-	26.86	2.86**	23.51	3.28*	17.13	1.54	32.38
A-level etc	-	22.69	3.25**	2.68	4.06*	15.47	1.41	19.05
Other higher education	-	15.06	3.06**	13.78	4.35*	12.15	1.02	1.48
Degree	-	29.58	5.02***	4.14	1.16***	54.14	2.34	32.38
Total	-	100	-	100	-	100	-	100
Household size								
(continues)	-	-	1.06	-	1.13*	-	1.08	-
Total	-	100	-	100	-	100	-	100
Constant	-	-	.27**	-	.05***	-	.07***	-

Note. RRR, relative risk ratio. Class 1 (cautiously negative) is the reference category.

3. Additional choice modelling results

This section presents additional results from various discrete choice models as referred to in the paper.

Table 5 shows the estimates the multinomial logit model in WTP space that provided the starting values for the MXL estimation. The alternative specific constant is negative and statistically significant, suggesting respondents are in principle less likely to select the opt-out alternative relative to one of the ATES deployment alternatives. They are willing to pay about £16 for the opportunity of private access to the system, and £23 for reducing CO2 emissions by 1000 tons. Increasing the time of the development by one year reduces utility by the equivalent of approximately £4. Finally, respondents value increasing their separation from the ATES installation at about £1.9.

Table 5. Multinomial logit estimates in WTP Space.

**Attribute**	**(1)<br>MNL**
Opt-out	-0.766***<br>(0.061)
Time to Completion	-3.707***<br>(0.258)
Private access	16.731***<br>(1.037)
CO2 emissions reduction	23.186***<br>(1.293)
Distance	1.859***<br>(0.369)
- Price	0.026***<br>(0.001)
Log-likelihood	-21503.02
AIC	43018.03
Individuals	1758
Observations	21096

Note. Estimates from a multinomial choice model in WTP space using data from the discrete choice experiment. Coefficient estimates on the non-monetary attributes capture the negative of mean marginal WTP in £. Time to completion, CO2 emissions reduction, Distance and Price are continuous. Private access is binary. AIC, Akaike Information Criterion. *** $p<0.01$.

Column 1 of Table 6 shows estimates from an MNL model in preference space. Estimates correspond to those presented in Table 5, since the model in WTP space is a reparameterisation of the model in preference space. Attribute coefficient estimates carry the expected signs.

Respondents appear to favour speedy deployment of ATES, as they are less likely to choose alternatives with greater time to completion. The positive coefficient on Private Access suggests respondents appreciate the opportunity to access the direct benefits of ATES installations for they own property's heating and cooling needs. All else equal, they are more likely to choose alternatives that imply greater CO2 emissions reduction, and alternatives proposing developments that are placed farther away from their properties. As predicted from economic theory, the coefficient on the price attribute is negative, suggesting that respondents are more likely to choose cheaper alternatives.

Table 6. Multinomial and Mixed Multinomial logit estimates in Preference Space.

Attribute	(1) Multinomial Logit	(2) Mixed Logit	
		Mean	St. Dev.
Option 3	-0.766***	-3.374***	4.283***
	(0.061)	(0.149)	(0.191)
Time to Completion	-0.098***	-0.157***	0.200***
	(0.006)	(0.010)	(0.014)
Private access	0.442***	0.554***	0.897***
	(0.022)	(0.032)	(0.043)
CO2 emissions reduction	0.613***	0.784***	0.936***
	(0.030)	(0.042)	0.058)
Distance	0.049***	0.076***	0.025
	(0.010)	(0.013)	(0.044)
Price	-0.026***	-3.828***	1.544***
	(0.001)	(0.078)	(0.066)
Log-likelihood	-21503.02	-15925.55	
AIC	43018.03	31875.11	
Individuals	1758	1758	
Observations	21096	21096	

Note. Column 1 presents estimates from a multinomial logit choice model in preference space using data from the discrete choice experiment. Column 2 shows estimates of the mean and standard deviation of the parameter distributions from mixed multinomial logit models. Time to completion, CO2 emissions reduction, Distance and Price are continuous. Private access is binary. Preferences for non-monetary attributes are normally distributed. The coefficient on price follows a negative log-normal distribution. AIC, Akaike Information Criterion. *** p<0.01

Column 2 of Table 6 reports estimates from a MXL model in preference space, letting the coefficients on the non-monetary attributes be normally distributed and the coefficient on price follow a negative log-normal distribution. Results align with the estimates from the MNL model in column 1, and the MXL models in WTP space reported in Table 1 of the paper. Respondents are positively inclined towards ATES installations that provide greater CO2 emissions reduction,

allow private households to access them, and are situated farther from their home. They are less likely to select more expensive alternatives, and options suggesting long development horizons.

Column 1 of Table 7 shows the implied WTP estimates from the MNL model in preference space along with their standard errors calculated using the Delta method. Estimates are as expected identical to those derived through the MNL model in WTP space reported in Table 7, and comparable to those derived from the MXL model in WTP space reported in the main text. Respondents are willing to pay about £3.7 to expedite ATES deployment by one year, £16 for securing private access to the system, £23 for reducing CO2 emissions by 1000 tons, and £1.88 for increasing the distance between their property and the ATES installation by 1 mile. Column 2 shows the mean and standard deviation of the WTP distribution for each attribute from the MXL model in preference space reported in Table 8, estimated using simulation. For the simulations we assume a constant coefficient on the price attribute to avoid draws from the tail of the log normal distribution that eventually lead to implausibly high mean WTP estimates. For this reason, estimates are not directly comparable to the ones in the main text. Mean WTP for expediting deployment by a year is about £7. Respondents are willing to pay about £26 for securing access to the system, £37 for decreasing CO2 emissions and £3.5 for increasing their separation from the installation by one mile.

Table 7. Willingness to pay for ATES deployment attributes.

**Attribute**	**(1)<br>MNL**	**(2)<br>MXL**
Time to Completion	3.70	-7.31
	(0.26)	(9.32)
Private access	16.73	25.82
	(1.04)	(41.81)
CO2 emissions reduction	23.19	36.54
	(1.29)	(43.63)
Distance	1.86	3.54
	(0.37)	(1.17)

Note: The table shows WTP estimates for each attribute. Column 1 shows average WTP and standard errors from the multinomial logit model presented in Table 6, calculated using the delta method. Column 2 shows the simulated mean and standard deviation of the WTP distribution for each of the attributes

Table 8 presents estimates from MNL and MXL models excluding 173 respondents that chose the opt-out alternative in each of the 12 choice sets. Estimates from the MXL are remarkably similar to those reported in Table 1 of the main text. In all cases respondents are more likely to choose an opt-in alternative relative to the opt-out. Respondents suffer a loss of utility equivalent to £3.5 from increasing the time to technology deployment by one year, are willing to pay £15 for having

the opportunity to access the system, £17 per 1000 tons of CO2 emissions reduction, and £1 to increase their distance from the ATES installation by 1km.

Table 8. MNL and MXL in WTP Space excluding serial objectors.

	(1)	(2)	
**Attribute**	**MNL**	**MXL**	
		**Mean**	**St. Dev.**
Option 3	-1.415***	-3.507***	2.551***
	(0.061)	(0.133)	(0.097)
Time to Completion	-3.871***	-3.590***	4.185***
	(0.262)	(0.288)	(0.405)
Private access	16.382***	15.062***	19.615***
	(0.982)	(1.051)	(1.101)
CO2 emissions reduction	22.699***	17.590***	16.902***
	(1.303)	(1.186)	(1.754)
Distance	1.628***	1.015***	1.268
	(0.377)	(0.297)	(0.799)
- Price	0.028***	3.416***	1.030***
	(0.001)	(0.050)	(0.047)
Log-likelihood	-17860.3	-15463.05	
AIC	35732.59	30950.09	
Individuals	1585	1585	
Observations	19020	19020	

Note: Column 1 shows estimates from an MNL model. Column 2 shows estimates of the mean and standard deviation of the WTP distributions from an MXL model when the non-monetary attributes follow a normal distribution and the price attribute follows a negative log-normal distribution. AIC, Akaike Information Criterion.

Table 9 shows estimates from MNL and MXL models in preference space when excluding respondents that consistently selected the opt out alternative. In all cases results are similar to those presented earlier and in the main text.

Table 9. MNL and MXL in Preference Space excluding serial objectors.

	(1)	(2)	(3)
**Attribute**	**MNL**	**MXL**	
		**Mean**	**St. Dev.**
Option 3	-1.415***	-3.409***	2.515***
	(0.061)	(0.125)	(0.097)
Time to Completion	-0.107***	-0.152***	0.193***
	(0.007)	(0.010)	(0.013)
Private access	0.454***	0.559***	0.864***
	(0.023)	(0.032)	(0.042)
CO2 emissions reduction	0.629***	0.794***	0.884***
	(0.032)	(0.042)	(0.056)
Distance	0.045***	0.074***	0.015

	(1)	(2)	
	(0.010)	(0.013)	(0.023)
Price	-0.028***	-3.787***	1.305***
	(0.001)	(0.077)	(0.079)
Log-likelihood	-17860.3	-15360.79	
AIC	35732.59	30745.59	
Individuals	1585	1585	
Observations	19020	19020	

Note: Column 1 shows estimates from an MNL model in preference space. Column 2 shows estimates of the mean and standard deviation of the parameter distributions for each attribute, from an MXL, assuming non-monetary attributes are normally distributed, and the price attribute is negative long-normal distributed. Standard errors in parentheses. AIC, Akaike Information Criterion. *** p<0.001.

Hybrid Choice Model

Table 10 shows estimates from the structural component of the HMXL model. Having completed higher education is positively related to more positive latent attitudes towards the systemic and local/individual benefits of ATES (LV1 and LV2 respectively), as well as the desire for more ATES-specific information (LV4), and to greater support for financial and political support. Household size is positively correlated to LV1, LV2 and LV5. Being in marriage is positively related to LV3, LV4 and LV5. Finally, being in full employment is negatively related to latent support for information and education.

Table 10. Structural component of the Hybrid MXL model.

	(1)<br>LV1	(2)<br>LV2	(3)<br>LV3	(4)<br>LV4	(5)<br>LV5
Female	-0.112	-0.067	0.065	0.022	-0.103
	(0.071)	(0.069)	(0.071)	(0.061)	(0.068)
Full Employment	0.084	0.065	0.119	-0.282***	0.082
	(0.064)	(0.057)	(0.071)	(0.067)	(0.069)
Higher Education	0.207***	0.266***	0.003	0.394***	0.206***
	(0.068)	(0.061)	(0.075)	(0.068)	(0.066)
In Marriage	0.065	0.083	0.167***	0.381***	0.186***
	(0.059)	(0.059)	(0.07)	(0.064)	(0.067)
Household Size	0.022***	0.028***	0.005	-0.030	0.044***
	(0.007)	(0.012)	(0.015)	(0.019)	(0.01)

Note: The table presents estimates from the structural component the Hybrid Choice MXL. The latent variables capture attitudes towards: Systemic benefits of ATES (column 1), Local and individual benefits of ATES (column 2), Safety and environmental concerns (column 3), Community engagement and education (column 4), Policy and financial support (column 5). Female, Full Employment, Higher education and In marriage are binary. Standard errors in parentheses.

Table 11 presents the measurement component of the HCM, capturing the relationship between the unobserved (latent) individual attitudes and the responses on the attitudinal questions posed to the respondents. The response to each attitudinal question is modelled as a function of a single latent variable. For instance, responses to indicators labelled *Sustainability, Benefits>Risks,* and *Safe UK use* are modelled as functions of Latent Variable 1. All estimated coefficients suggest that individual responses to the attitudinal questions are positively correlated with the corresponding latent variables. This suggests for example that, individuals who agree more strongly with statements “ATES can be a viable and sustainable solution for long-term energy storage and the stability of electricity supply”, “I believe that the benefits of ATES, such as energy cost savings and reduced dependence on fossil fuels, outweigh any potential risks” and “I believe that ATES deserves a try and should be deployed where appropriate and safe to do so in the UK”, have more positive attitudes towards the systemic benefits of ATES deployment.

Table 11. Measurement component of the Hybrid Choice Model.

	(1)	(2)	(3)	(4)	(5)
	**LV 1**	**LV2**	**LV3**	**LV4**	**LV5**
Sustainability	2.908***				
	(0.162)				
Benefits> Risks	2.673***				
	(0.145)				
Safe UK use	3.074***				
	(0.188)				
Local benefit		2.994***			
		(0.18)			
Open use		2.707***			
		(0.164)			
Community OK		3.387***			
		(0.235)			
Reliability			1.512***		
			(0.106)		
Contamination risk			1.901***		
			(0.13)		

Safety	1.833***		
	(0.131)		
Education		1.800***	
		(0.128)	
Join talk		0.844***	
		(0.07)	
Input needed		1.631***	
		(0.106)	
Funding			1.799***
			(0.121)
Pay support			1.988***
			(0.12)
Incentives			2.608***
			(0.209)

Sustainability: ATES can be a viable and sustainable solution for long-term energy storage and the stability of electricity supply. *Benefits > Risks:* I believe that the benefits of ATES, such as energy cost savings and reduced dependence on fossil fuels, outweigh any potential risks. *Safe UK use:* I believe that ATES deserves a try and should be deployed where appropriate and safe to do so in the UK. *Local benefit:* I believe that ATES technology has the potential to benefit my local area in terms of energy efficiency and sustainability. *Open use:* I am receptive to using ATES as a heating and cooling solution for buildings in my neighbourhood. *Community OK:* I am open to the idea of having ATES systems installed within my community. *Reliability:* I have reservations about the long-term reliability and performance of ATES systems. *Contamination risk:* I believe there is a risk that ATES could contaminate groundwater and drinking water. *Safety:* I am concerned about the safety of ATES, such as subsidence, fracking, groundwater contamination, and thermal pollution. *Education:* I think that public awareness and education about ATES should be increased to address any misconceptions or concerns. *Join talk:* I am willing to participate in community discussions and decision-making processes regarding the implementation of ATES projects at the local level. *Input needed:* I believe that ATES projects should undergo thorough environmental assessments and community consultations before being implemented at the local level. *Funding:* I think it is necessary for local governments to collaborate with external funding sources, such as regional or national agencies, to secure financial support for ATES initiatives. *Pay support:* I am willing to contribute a small surcharge or fee to support a local fund dedicated to providing financial support for ATES projects in our community. *Incentives:* I am in favour of local government incentives or policies to encourage the adoption of ATES technology in my community.

Hybrid choice model without serial objectors

Tables 12-14 show estimates from the choice, structural and measurement components of a HMXL excluding individuals consistently selecting the opt-out alternative.

Table 12. Choice component of the Hybrid MXL model excluding serial objectors.

	(1)		(2)	(3)	(4)	(5)	(6)
	**Mean**	**St. Dev.**	**LV 1**	**LV2**	**LV3**	**LV4**	**LV5**
Option 3	-2.811***	2.193***	-0.665**	-0.860**	-0.068	0.952***	-0.396
	(0.238)	(0.163)	(0.293)	(0.403)	(0.276)	(0.320)	(0.269)

Time to completion	-3.592***	4.230***	0.093	-1.500***	0.185	0.045	-0.232
	(0.372)	(0.551)	(1.278)	(0.537)	(0.334)	(0.348)	(0.595)
Private access	13.832***	16.441***	-0.055	6.474**	-2.398	2.022	0.783
	(1.893)	(1.454)	(2.121)	(2.533)	(1.556)	(4.449)	(2.848)
CO_2 emissions reduction	17.503***	10.005*	-3.595	5.844	-5.560	7.726	-0.925
	(5.407)	(5.238)	(6.271)	(6.64)	(4.469)	(8.048)	(9.907)
Distance	1.305	0.300	-1.451	0.125	0.566	1.574*	0.061
	(0.846)	(0.912)	(1.862)	(1.773)	(0.825)	(0.834)	(1.421)
- Price	3.429***	0.806***	-0.037	0.194**	-0.032	-0.273*	0.085
	(0.082)	(0.151)	(0.122)	(0.093)	(0.104)	(0.146)	(0.113)
Log-likelihood				-43756.42			
AIC				87796.84			
Individuals				1585			
Observations				19020			

Note: The table presents estimates from the choice component from a Hybrid Choice MXL in WTP space. The dependent variable is in all cases respondent choice between three profiles of ATES deployment. Option 3 is the opt-out alternative. Time to completion is measured in years. Private access is binary, equal to 1 if the ATES deployment permits private household connections. CO2 emissions reduction is measured in thousands of tons. Distance is measured in meters and price in GBP. Columns 1 and 2 show estimates of the mean and standard deviation (SD) of the WTP distributions for each attribute. Columns 2-6 show estimates of the coefficients on the interaction terms between the alternative specific constant and each of the latent variables. The latent variables capture attitudes towards: Systemic benefits of ATES (column 2), Local and individual benefits of ATES (column 3), Safety and environmental concerns (column 4), Community engagement and education (column 5), Policy and financial support (column 6). Standard errors in parentheses. AIC: Akaike Information Criterion. Models are estimated using 5000 Sobol draws.

Table 13. Structural component of the Hybrid MXL model excluding serial objectors.

	(1)<br>LV1	(2)<br>LV2	(3)<br>LV3	(4)<br>LV4	(5)<br>LV5
Female	-0.121*	-0.177**	0.209***	-0.016	-0.179**
	(0.072)	(0.082)	(0.072)	(0.088)	(0.089)
Full Employment	-0.029**	0.026	0.121	-0.237***	0.025
	(0.091)	(0.069)	(0.079)	(0.086)	(0.139)
Higher Education	0.201	0.243***	0.037**	0.374***	0.204***
	(0.095)	(0.075)	(0.079)	(0.074)	(0.070)
In Marriage	0.103	0.156*	0.155	0.432***	0.258***
	(0.078)	(0.083)	(0.149)	(0.097)	(0.083)

Household Size	0.019	0.037***	0.029	-0.051***	0.027**
	(0.015)	(0.012)	(0.015)	(0.018)	(0.012)

Note: The table presents estimates from the structural component the Hybrid Choice MXL. The latent variables capture attitudes towards: Systemic benefits of ATES (column 1), Local and individual benefits of ATES (column 2), Safety and environmental concerns (column 3), Community engagement and education (column 4), Policy and financial support (column 5). Female, Full Employment, Higher education and In marriage are binary. Standard errors in parentheses. *** p<0.01, ** p<0.05, * p<0.1

Table 14. Measurement component of the Hybrid Choice Model excluding serial objectors.

	**(1)**	**(2)**	**(3)**	**(4)**	**(5)**
	**LV 1**	**LV2**	**LV3**	**LV4**	**LV5**
Sustainability	2.769***				
	(0.188)				
Benefits> Risks	2.283***				
	(0.144)				
Safe UK use	2.612***				
	(0.189)				
Local benefit		2.566***			
		(0.165)			
Open use		2.266***			
		(0.179)			
Community OK		2.802***			
		(0.289)			
Reliability			1.396***		
			(0.124)		
Contamination risk			1.954***		
			(0.184)		
Safety			1.756***		
			(0.144)		
Education				1.975***	
				(0.172)	
Join talk				0.784***	
				(0.073)	
Input needed				1.754***	
				(0.137)	
Funding					1.717***
					(0.189)
Pay support					1.667***

	(0.147)
Incentives	2.515^{***}
	(0.358)

Sustainability: ATES can be a viable and sustainable solution for long-term energy storage and the stability of electricity supply. *Benefits > Risks:* I believe that the benefits of ATES, such as energy cost savings and reduced dependence on fossil fuels, outweigh any potential risks. *Safe UK use:* I believe that ATES deserves a try and should be deployed where appropriate and safe to do so in the UK. *Local benefit:* I believe that ATES technology has the potential to benefit my local area in terms of energy efficiency and sustainability. *Open use:* I am receptive to using ATES as a heating and cooling solution for buildings in my neighbourhood. *Community OK:* I am open to the idea of having ATES systems installed within my community. *Reliability:* I have reservations about the long-term reliability and performance of ATES systems. *Contamination risk:* I believe there is a risk that ATES could contaminate groundwater and drinking water. *Safety:* I am concerned about the safety of ATES, such as subsidence, fracking, groundwater contamination, and thermal pollution. *Education:* I think that public awareness and education about ATES should be increased to address any misconceptions or concerns. *Join talk:* I am willing to participate in community discussions and decision-making processes regarding the implementation of ATES projects at the local level. *Input needed:* I believe that ATES projects should undergo thorough environmental assessments and community consultations before being implemented at the local level. *Funding:* I think it is necessary for local governments to collaborate with external funding sources, such as regional or national agencies, to secure financial support for ATES initiatives. *Pay support:* I am willing to contribute a small surcharge or fee to support a local fund dedicated to providing financial support for ATES projects in our community. *Incentives:* I am in favour of local government incentives or policies to encourage the adoption of ATES technology in my community.

Table 15. Measures of attitudes toward ATES.

Variable name	Statement about Aquifer Thermal Energy Storage	Latent Attitudes
Sustainability	ATES can be a viable and sustainable solution for long-term energy storage and the stability of electricity supply	System level impact
Reliability	I have reservations about the long-term reliability and performance of ATES systems.	Safety and environmental concerns
Education	I think that public awareness and education about ATES should be increased to address any misconceptions or concerns.	Community engagement and education
Benefits > Risks	I believe that the benefits of ATES, such as energy cost savings and reduced dependence on fossil fuels, outweigh any potential risks.	System level impact
Safe UK use	I believe that ATES deserves a try and should be deployed where appropriate and safe to do so in the UK.	System level impact
Local benefit	I believe that ATES technology has the potential to benefit my local area in terms of energy efficiency and sustainability.	Local and individual use and contribution
Contamination risk	I believe there is a risk that ATES could contaminate groundwater and drinking water.	Safety and environmental concerns
Open use	I am receptive to using ATES as a heating and cooling solution for buildings in my neighbourhood.	Local and individual use and contribution
Community OK	I am open to the idea of having ATES systems installed within my community.	Local and individual use and contribution
Funding	I think it is necessary for local governments to collaborate with external funding sources, such as regional or	Policy and financial support

	national agencies, to secure financial support for ATES initiatives.	
Pay support	I am willing to contribute a small surcharge or fee to support a local fund dedicated to providing financial support for ATES projects in our community.	Policy and financial support
Incentives	I am in favour of local government incentives or policies to encourage the adoption of ATES technology in my community.	Policy and financial support
Join talk	I am willing to participate in community discussions and decision-making processes regarding the implementation of ATES projects at the local level.	Community engagement and education
Safety	I am concerned about the safety of ATES, such as subsidence, fracking, groundwater contamination, and thermal pollution.	Safety and environmental concerns
Input needed	I believe that ATES projects should undergo thorough environmental assessments and community consultations before being implemented at the local level.	Community engagement and education

Note. The table presents the attitudinal questions respondents were called to answer. Responses were given on a scale from (1 = Strongly disagree to 5 = Strongly agree).

Robustness tests

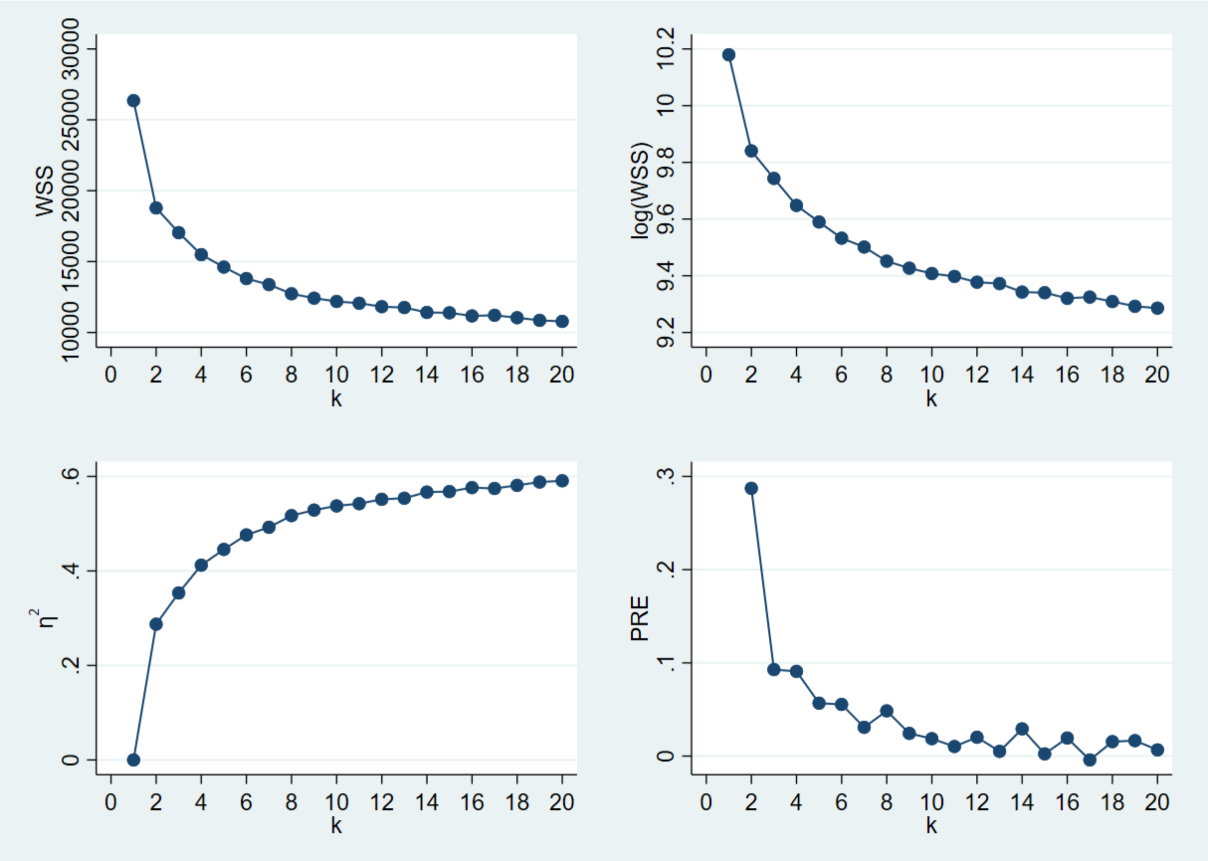

Figure 2. WSS, log (WSS), η 2, and PRE for all k-cluster solutions of ATES attitudes.

Note. WSS (within sum of squares), PRE (proportional reduction of error). k stands for the number of clusters.

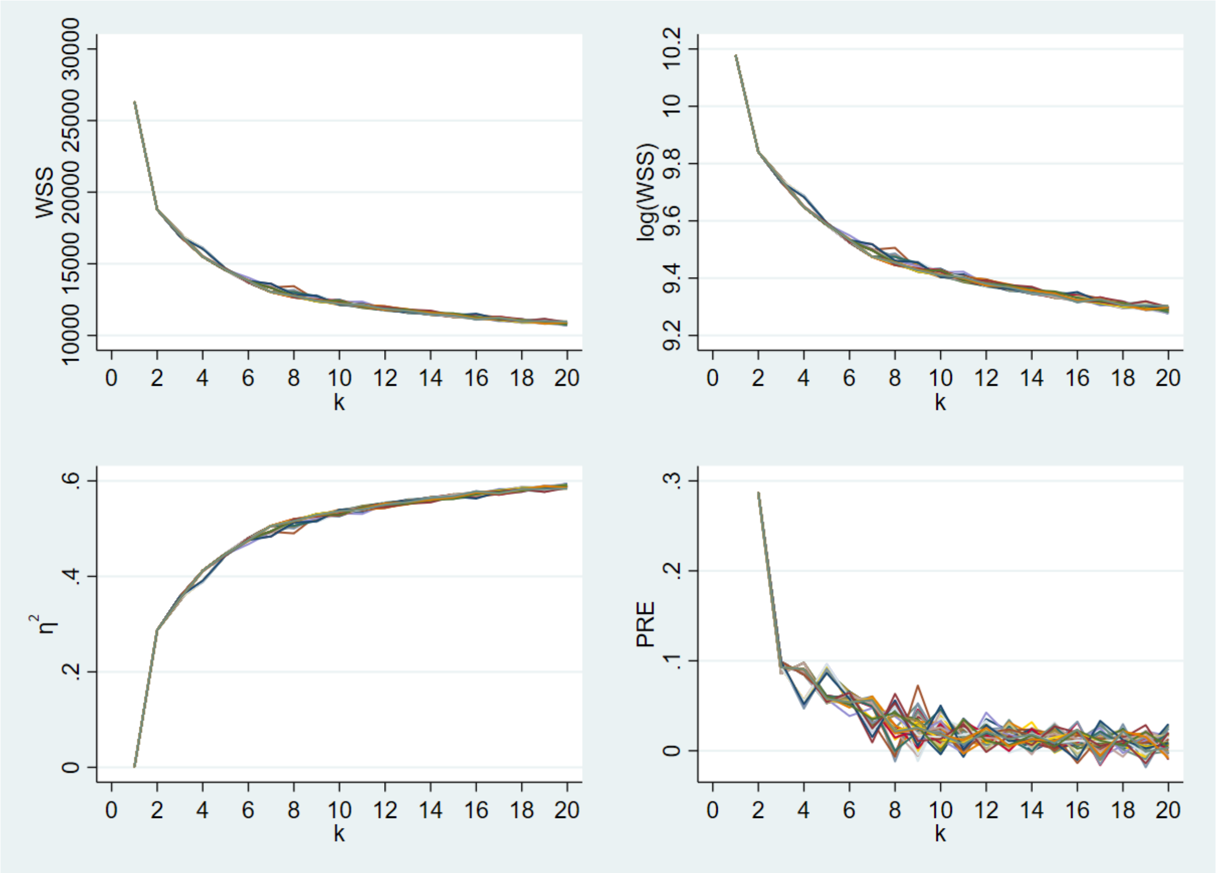

Figure 3. Fifty different WSS, log (WSS), η^2, and PRE for all k-cluster solutions of ATES attitudes. *Note.* WSS (within sum of squares), PRE (proportional reduction of error). k stands for the number of

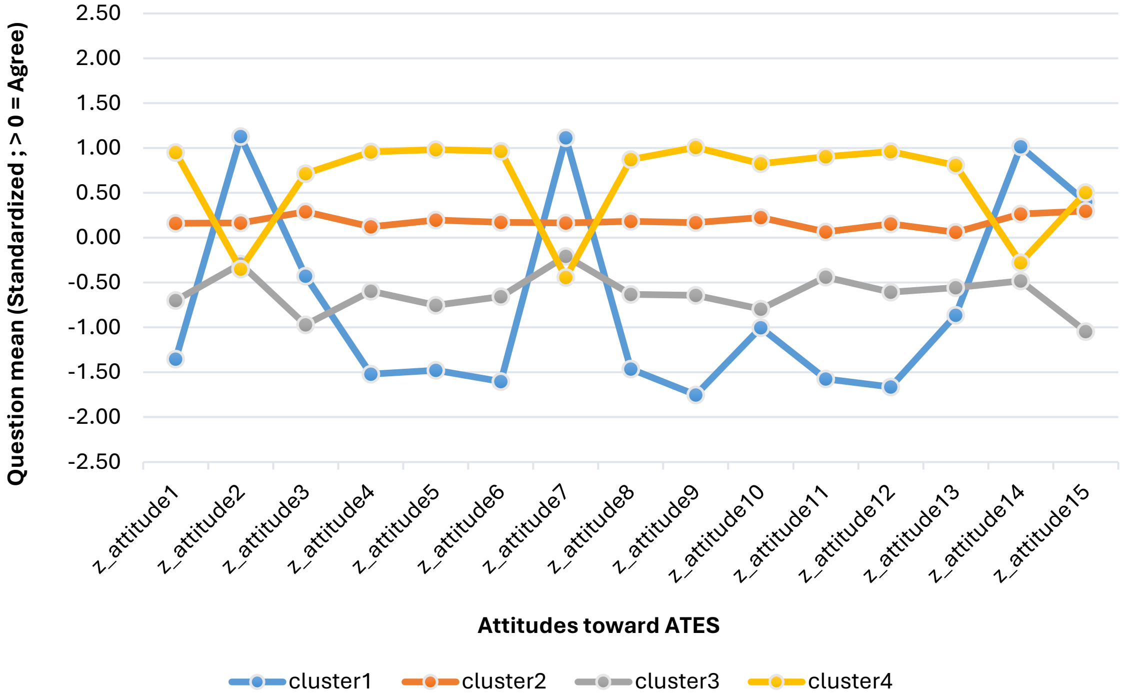

clusters.

Figure 4. Item means for the four -cluster ATES attitudes model using K-means clustering.

Figure 5: An example of a choice card from the DCE.

	**Option 1**	**Option 2**	**Option 3**
**Time to deployment**	4 years	2 years	No Aquifer Thermal Energy Storage
**Private household access**	Yes	No	
**CO_2 Emissions Reduction**	150 tons	1000 tons	
**Distance to nearest installation**	100 meters	1 km	
**One-off Cost**	£15	£20	
**Your Choice**			
