# Supplementary material for: Heterogeneity in public attitudes and preferences for the deployment of aquifer thermal energy storage
Source: Nat Energy. 2026 Mar 6;11(3):479–89. doi: 10.1038/s41560-026-01977-z (PMC13021514; doi:10.1038/s41560-026-01977-z)
Supplement: Supplementary file 2 — Reporting Summary [file 41560_2026_1977_MOESM2_ESM.pdf]

nature portfolio

Corresponding author(s): Yiannis Kountouris

Last updated by author(s): Dec 26, 2025

Reporting Summary

Nature Portfolio wishes to improve the reproducibility of the work that we publish. This form provides structure for consistency and transparency in reporting. For further information on Nature Portfolio policies, see our Editorial Policies and the Editorial Policy Checklist.

Statistics

For all statistical analyses, confirm that the following items are present in the figure legend, table legend, main text, or Methods section.

n/a	Confirmed	
☐	☒	The exact sample size (*n*) for each experimental group/condition, given as a discrete number and unit of measurement
☒	☐	A statement on whether measurements were taken from distinct samples or whether the same sample was measured repeatedly
☐	☒	The statistical test(s) used AND whether they are one- or two-sided<br>*Only common tests should be described solely by name; describe more complex techniques in the Methods section.*
☐	☒	A description of all covariates tested
☐	☒	A description of any assumptions or corrections, such as tests of normality and adjustment for multiple comparisons
☐	☒	A full description of the statistical parameters including central tendency (e.g. means) or other basic estimates (e.g. regression coefficient) AND variation (e.g. standard deviation) or associated estimates of uncertainty (e.g. confidence intervals)
☐	☒	For null hypothesis testing, the test statistic (e.g. *F*, *t*, *r*) with confidence intervals, effect sizes, degrees of freedom and *P* value noted<br>*Give P values as exact values whenever suitable.*
☒	☐	For Bayesian analysis, information on the choice of priors and Markov chain Monte Carlo settings
☒	☐	For hierarchical and complex designs, identification of the appropriate level for tests and full reporting of outcomes
☒	☐	Estimates of effect sizes (e.g. Cohen's *d*, Pearson's *r*), indicating how they were calculated

Our web collection on statistics for biologists contains articles on many of the points above.

Software and code

Policy information about availability of computer code

Data collection	The social survey data were collected in the United Kingdom using the Qualtrics online survey platform. The survey included socio-demographic questions, items assessing attitudes toward and awareness of Aquifer Thermal Energy Storage (ATES), and a discrete choice experiment designed to elicit respondents' preferences and willingness to pay (WTP) for various attributes related to ATES.
Data analysis	The Latent Class Analysis was performed on Stata 18. Multinomial Logit Models, Mixed Multinomial Logit Models and Hybrid Choice Models were estimated in R using the Apollo Package version 0.3.6. We used the Imperial College HPC facility for model estimation.

For manuscripts utilizing custom algorithms or software that are central to the research but not yet described in published literature, software must be made available to editors and reviewers. We strongly encourage code deposition in a community repository (e.g. GitHub). See the Nature Portfolio guidelines for submitting code & software for further information.

Data

Policy information about availability of data

All manuscripts must include a data availability statement. This statement should provide the following information, where applicable:

- Accession codes, unique identifiers, or web links for publicly available datasets
- A description of any restrictions on data availability
- For clinical datasets or third party data, please ensure that the statement adheres to our policy

The data are available online at https://doi.org/10.6084/m9.figshare.30762362.

Research involving human participants, their data, or biological material

Policy information about studies with human participants or human data. See also policy information about sex, gender (identity/presentation), and sexual orientation and race, ethnicity and racism.

Reporting on sex and gender	Gender information was collected as part of the socio-demographic section of the survey. Respondents were asked to indicate their gender identity (e.g., male, female, non-binary, prefer not to say), and this variable was used in the analysis to explore potential differences in attitudes, preferences and willingness to pay (WTP) across gender groups. Interaction effects involving gender are reported where statistically meaningful. We acknowledge the importance of inclusive and accurate representation of gender in research.
Reporting on race, ethnicity, or other socially relevant groupings	Information on race, ethnicity, or other socially relevant groupings was not collected in this study. The decision was based on the focus of the research, which centered on socio-demographic factors such as gender, education, employment, household characteristics, and attitudes toward sustainable energy technologies. While the survey was conducted in the UK, and regional representativeness was considered in sampling, we acknowledge that race and ethnicity are important dimensions of social identity that may influence perceptions and preferences. Future research could incorporate such variables to better understand equity implications in the adoption of low-carbon technologies.
Population characteristics	The research sample consisted of adults residing in the Northwest of England, UK. A total of 1,758 respondents participated in the survey. Of these, 53.7% identified as female and 45.7% as male. Nearly 40% of participants were between the ages of 25 and 44. Just over half (52.9%) had attained at least a higher education qualification. Approximately 48.9% were employed full-time, and a similar proportion (48.8%) were married. The majority of respondents (about 89%) lived in households with fewer than five members.
Recruitment	Respondents were recruited from a panel provided by Norstat, a major third-party survey recruitment company. Respondents were selected via quota sampling with regionally representative quotas for location, gender, age, and level of education.
Ethics oversight	Imperial College Ethics Board (SETREC number: 6799602)

Note that full information on the approval of the study protocol must also be provided in the manuscript.

Field-specific reporting

Please select the one below that is the best fit for your research. If you are not sure, read the appropriate sections before making your selection.

☐ Life sciences ☒ Behavioural & social sciences ☐ Ecological, evolutionary & environmental sciences

For a reference copy of the document with all sections, see nature.com/documents/nr-reporting-summary-flat.pdf

Behavioural & social sciences study design

All studies must disclose on these points even when the disclosure is negative.

Study description	Our study employed a quantitative approach, using a social survey to gather data on public attitudes and preferences regarding the deployment of ATES in public buildings in the UK. Drawing on responses from both the survey and a discrete choice experiment, we find substantial heterogeneity in public attitudes and levels of support for ATES installations.
Research sample	The study sample consisted of adult residents of Northwest of England, UK. Quotas were applied to achieve a broadly representative and balanced-sample in terms of age, gender, and location. The final sample included respondents from diverse employment statuses, education levels, household sizes, and marital statuses.
Sampling strategy	The survey was administered online, with IP address targeting and a residential location question used to focus on respondents from the Northwest of England. The study concentrated on this region due to its potential for deploying Aquifer Thermal Energy Storage. Our analysis was limited to adults living in council tax-paying households, as the choice experiment included a hypothetical scenario in which ATES systems could be connected to private homes, affecting local tax contributions. Participants were selected through quota sampling to ensure regional representativeness based on location, gender, age, and education level.
Data collection	Data were collected through a survey instrument designed to elicit information about individual attitudes and preferences for the deployment of ATES in the Northwest of England.
Timing	Data were collected between 25 June 2024 and 22 July 2024
Data exclusions	Respondents under the age of 18 were excluded. Only respondents that fully completed the survey were included in the sample.
Non-participation	No participants dropped out of the analysis
Randomization	Participants were not allocated to experimental groups

Reporting for specific materials, systems and methods

We require information from authors about some types of materials, experimental systems and methods used in many studies. Here, indicate whether each material, system or method listed is relevant to your study. If you are not sure if a list item applies to your research, read the appropriate section before selecting a response.

Materials & experimental systems

n/a	Involved in the study
☒	☐ Antibodies
☒	☐ Eukaryotic cell lines
☒	☐ Palaeontology and archaeology
☒	☐ Animals and other organisms
☒	☐ Clinical data
☒	☐ Dual use research of concern
☒	☐ Plants

Methods

n/a	Involved in the study
☒	☐ ChIP-seq
☒	☐ Flow cytometry
☒	☐ MRI-based neuroimaging

Plants

Seed stocks

Report on the source of all seed stocks or other plant material used. If applicable, state the seed stock centre and catalogue number. If plant specimens were collected from the field, describe the collection location, date and sampling procedures.

Novel plant genotypes

Describe the methods by which all novel plant genotypes were produced. This includes those generated by transgenic approaches, gene editing, chemical/radiation-based mutagenesis and hybridization. For transgenic lines, describe the transformation method, the number of independent lines analyzed and the generation upon which experiments were performed. For gene-edited lines, describe the editor used, the endogenous sequence targeted for editing, the targeting guide RNA sequence (if applicable) and how the editor was applied.

Authentication

Describe any authentication procedures for each seed stock used or novel genotype generated. Describe any experiments used to assess the effect of a mutation and, where applicable, how potential secondary effects (e.g. second site T-DNA insertions, mosiacism, off-target gene editing) were examined.
